# A case of membranoproliferative glomerulonephritis associated withcurved fibril deposition

**DOI:** 10.1186/s12882-015-0147-9

**Published:** 2015-09-15

**Authors:** Ikuyo Narita, Michiko Shimada, Takeshi Fujita, Reiichi Murakami, Masayuki Nakamura, Norio Nakamura, Hideaki Yamabe, Ken Okumura

**Affiliations:** Department of Cardiology and Nephrology, Hirosaki University Graduate School of Medicine, 5 Zaifu-cho, Hirosaki, 036-8562 Japan; Community Medicine, Hirosaki University Graduate School of Medicine, 5 Zaifu-cho, Hirosaki, 036-8562 Japan

**Keywords:** Membranoproliferative glomerulonephritis, Fibrillary glomerulonephritis, Immunotactoid glomerulopathy

## Abstract

**Background:**

It is sometimes challenging to diagnose unsusual cases of fibrillary glomerulonephritis (FGN) and immunotactoid glomerulopathy (ITG), the rare causes of nephrotic syndrome.

**Case presentation:**

A 75-year-old Japanese woman presented with nephrotic syndrome, microhematuria and renal insufficiency. Renal biopsy revealed membranoproliferative glomerulonephritis (MPGN) with IgM and weak C3 deposition. Congo red stain was negative. Electron microscopy demonstrated massive fibrils in the subendothelium, mesangium and subepithelium. The fibrils were partially parallel, partially curved and 17 nm in diameter. Cryoglobulin, hepatitis B virus (HBV) antigen, hepatitis C virus (HCV) antibody or antinuclear antibody were negative.

**Conclusion:**

We report a case of MPGN associated with peculiar non-amyloid fibril deposition corresponding to neither FGN nor ITG.

## Background

FGN and ITG are glomerular deposition diseases with immunoglobulin-derived non-amyloid fibrils. The distinction between ITG and FGN is generally based on the appearance, thickness and the arrangement of the deposited fibrils. Typically, in FGN, fibrils are solid, 12–24 nm in diameter and the arrangement is random. Whereas, in ITG, fibrils are microtubule, diameter is >30 nm and parallel arrangement is observed [1].

In this report, we describe a case of MPGN associated with peculiar fibril deposits corresponding to neither FGN nor ITG.

## Case presentation

A 75-year-old Japanese woman presented with nephrotic syndrome, microhematuria and renal insufficiency. She had been treated as hypertension for 15 years. She was deaf since she was 6 years old for unknown reason. 6 months prior to admission, she developed erythema nodosum but Crohn’s disease, Sarcoidosis, Mycobacterium tuberculosis and malignancy were denied, and had been treated with prednisolone 10 mg per day. Then she noted pretibial edema and was referred to our hospital as nephrotic syndrome.

**Fig. 1:**
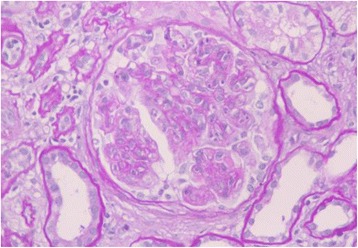
Light microscopy showed lobular accentuation and mesangial hypercellularity

**Fig. 2:**
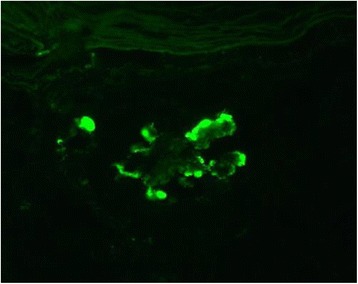
Immunofluorescent staining for IgM was positive along the capillary loop

**Fig. 3:**
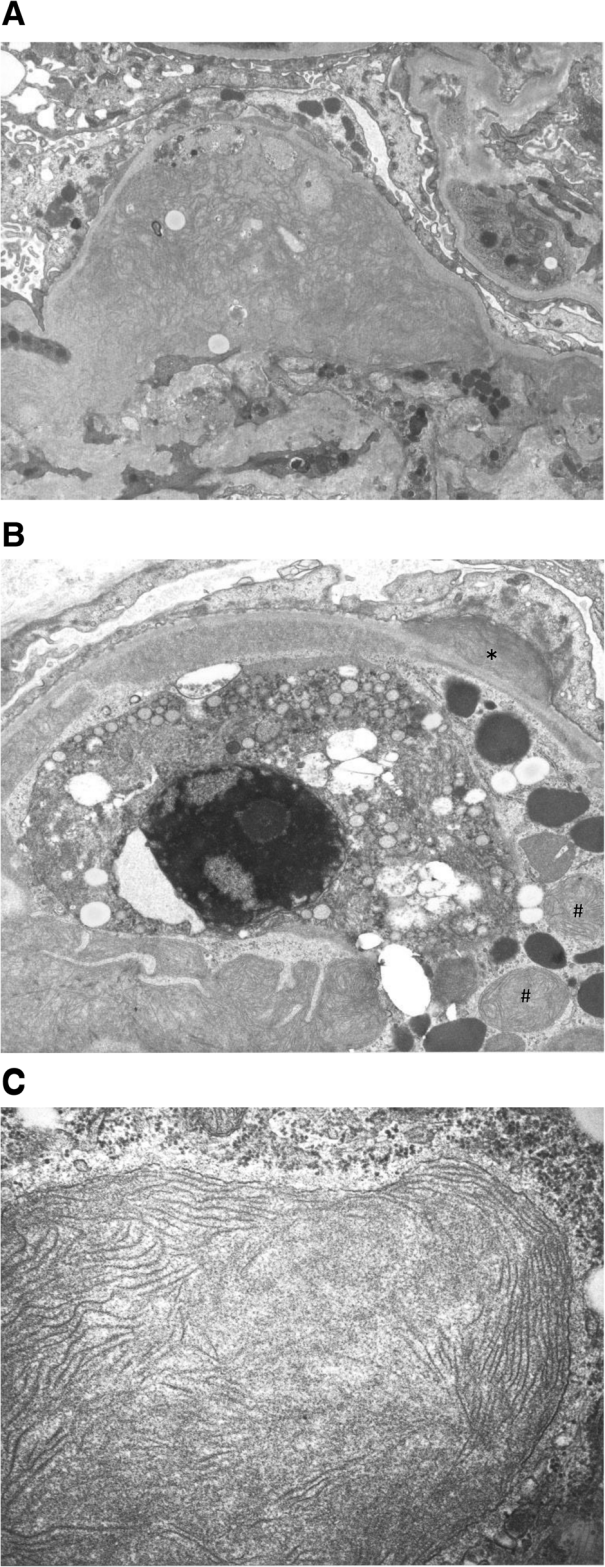
**a** Electron microscopy showed effacement of the glomerular epithelial foot process and fibrilar deposits in the subendothelial and mesangial area (original magnification × 5750). **b** Massive deposits are present in the mesangial and subendothelial area, and some of them formed spherical structure (#). Subepithelial deposit is also observed (*) (original magnification × 11500). **c** The deposits consist of non-branching elongated fibrils. The fibrils are partially parallel and 17 nm in diameter (original magnification × 46000)

At the time of admission, blood pressure was 177/99 mmHg, the height was 148 cm, and the weight was 58.4 kg. She exhibited edema in her face and both lower extremities. Pigmentation was seen on her legs but erythema was not present. The results of laboratory test on admission were as follows: hemoglobin 9.4 g/dl; red blood count 336 × 104/μl; white cell count 11500/μl; platelet count 569 × 103/μl; blood urea nitrogen 18 mg/dl; serum creatinine (sCr) 1.55 mg/dl; serum total cholesterol 286 mg/dl; triglycerides, 249 mg/dl; total protein 4.9 g/dl; albumin 2.3 g/dl; serum IgG 301 mg/dl; IgA 42 mg/dl; IgM 28 mg/dl. The levels of serum complement, free κ and λ light chains were within normal limits. Electrophoresis of serum and urine revealed no monoclonal spikes. Cryoglobulin, HBV antigen, HCV antibody and antinuclear antibody were not detected. Urinalysis showed massive proteinuria (8.0 g/gCr) and microscopic hematuria (>100/HPF). A renal biopsy was performed. On light microscopy, 13 glomeruli were observed and 11 glomeruli exhibited global sclerosis and two glomeruli exhibited lobular accentuation and mesangial hypercellularity, therefore, diagnosed as membranoproliferative glomerulonephritis (MPGN) (Fig. 1). Immunofluorescent study showed weakly positive C3 and fringe pattern for IgM (Fig. 2). Whereas, staining for IgA, IgG and C1q were negative. Congo red staining was negative. Additional immunefluorescent staining for IgG1-4, kappa light chain, delta light chain were also negative. Electron microscopy showed effacement of the glomerular epithelial foot process and massive fibril deposits in the subendothelium, mesangial area and subepithelium (Fig. 3a, b). Under high magnification, the fibrils were non-branching, partially parallel, partially curved and 17 nm in diameter, and some of the fibrils formed spherical structure (Fig. 3c). Additionally, bone marrow biopsy was performed and no specific abnormalities were found. She was treated with increased dose of oral predonisolone (20 mg/day). In 3 month, the amount of urinary protein was reduced from 8.0 to 2.0 g/gCr, however, the level of sCr increased from 1.5 to 2.5 mg/dl. The amount of urinary protein was re-increased concomitant with the reduction of predonisolone. Whereas, serum creatinine levels were progressively elevated, and she required hemodialysis in 15 months since the renal biopsy.

## Discussion

In the glomerulopathies with congo-red negative fibrillar deposits, firstly, non-immunoglobulin derived deposits in diabetes mellitus needs to be excluded. Next, lupus nephritis and cyroglobulinemia should be excluded [1]. Some experts contend that monoclonal gammopathy as well needs to be excluded before the diagnosis of FGN and ITG [1, 2], although some of the previous case reports included the cases with monoclonal spikes in the serum or in the urine [3, 4]. The diagnosis of FGN and ITG has been relatively established in the typical cases, however, there always has been some overlap [5], and fibrils sometimes have atypical appearances, for example, they may be curved or have lamellar structure [4, 6]. In our case, the fibrils were partially parallel, partially curved and appearing as 17 nm in diameter. The fibrils did not have the microtubular appearances, and some of the fibrils formed spherical structures which are the rare findings [7]. Thus, the fibrils were not compatible with typical FGN nor ITG.

In our case, IgM and C3 staining was positive, but IgG was negative. The Brady et al. analyzed the results of immunofluorescence data in the FGN or ITG. IgG was positive in 97 % cases, and IgM was positive in ~50 % [8]. The absence of IgG is unusual, however, it was probably because most glomeruli already reached total sclerosis which hampered proper staining. The diagnosis of MPGN in the light microscopy was most frequent in FGN or ITG in the past report [9], thus our case was compatible.

Pronovost et al. analyzed 186 patients with FGN/ITG, and suggested that lymphoproliferative malignancy was more frequent in ITG compared with FGN, although the significance disappeared when the patients with monoclonal spike in the urine or in the serum were excluded [5]. In the comparison of 61 patients with FGN and 6 patients with ITG, Rosenstock et al. reported that lymphoproliferative disease, monoclonal spike in the serum or urine and hypocomplementamia was significantly frequent in ITG [9]. Of note, they reported a case initially diagnosed as ITG, and cryogloblinemia was detected in the third attempt. In the cases with HCV positive or hypocomplementemia, repeated tests for the cryogloblinemia may be necessary.

Generally, renal prognosis is poor and progression to end stage renal disease (ESRD) occurs in approximately half of the patients within several years [4]. In our case, proteinuria was partially responsive to corticosteroid, however, renal dysfunction was rapidly progressive and reached ESRD in 15 months. The previous reports also showed the effectiveness of the corticosteroid for the treatment of FGN or ITG in some cases [10, 11].

The precise pathogenesis of FGN and ITG is still unknown, and the differences in the clinical courses are obscure. Therefore, there still is a debate whether FGN and ITG are two separate conditions or not.

In contrast to Amyloidosis, systemic involvement is unusual in the FGN/ITG. In our case, erythematosus nodosum appeared 6 months prior to the diagnosis of nephrotic syndrome, however, the causal relationship was unclear.

Thus, in the present case, the clinical course, pathological findings in PAS staining and immunofluorescent staining were compatible with FGN or ITG, although morphological findings in the electron microscopy were applicable to neither FGN nor ITG.

## Conclusions

In conclusion, electron microscopy is essential for the diagnosis of glomerulopathy with fibril depositions. We report a peculiar case of MPGN associated with nonspecific fibril deposition which applies to neither FGN nor ITG.

## Consent

Written informed consent was obtained from the patient for publication of the study in the BMC Nephrology.

## Abbreviations

FGN: Fibrillary glomerulonephritis
ITG: Immunotactoid glomerulopathy
MPGN: Membranoproliferative glomerulonephritis
HBV: Hepatitis B virus
HCV: Hepatitis C virus
ESRD: End stage renal disease
